# Supplementary material for: Hybrid Models and Biological Model Reduction with PyDSTool
Source: PLoS Comput Biol. 2012 Aug 9;8(8):e1002628. doi: 10.1371/journal.pcbi.1002628 (PMC3415397; doi:10.1371/journal.pcbi.1002628)
Supplement: Text S4 — Complete source code for the PyDSTool package (version 0.88.120504). Includes API documentation and help files linking to web pages. This file is identical to the current public release on Sourceforge.net. (ZIP) [file pcbi.1002628.s004.zip › PyDSTool/html/PyDSTool.MProject.dsInterface-class.html]

xml version="1.0" encoding="ascii"?


PyDSTool.MProject.dsInterface


| Home | Trees | Indices | Help | PyDSTool |
| --- | --- | --- | --- | --- |

Package PyDSTool ::
Module MProject ::
Class dsInterface

[hide private]

[frames] | no frames]

# Class dsInterface

source code

```
object --+
         |
        dsInterface
```

Known Subclasses:
:   - GeneratorInterface
    - , ModelInterface

---

Generic and abstract interface class for dynamical systems.


|  |  |
| --- | --- |
| Instance Methods | [hide private] |

|  |  |
| --- | --- |
| get\_test\_traj(self) | source code |

|  |  |
| --- | --- |
| query(self, querykey=`'``'`) | source code |

**Inherited from `object`**:
`__delattr__`,
`__getattribute__`,
`__hash__`,
`__init__`,
`__new__`,
`__reduce__`,
`__reduce_ex__`,
`__repr__`,
`__setattr__`,
`__str__`


|  |  |
| --- | --- |
| Class Variables | [hide private] |

\_setkeys = `['globalt0', 'tdata', 'pars', 'algparams', 'inputs']`

\_querykeys = `['pars', 'parameters', 'events', 'submodels', 'ic...`


|  |  |
| --- | --- |
| Properties | [hide private] |

**Inherited from `object`**:
`__class__`


|  |  |
| --- | --- |
| Class Variable Details | [hide private] |

### \_querykeys

Value:
:   ```
    ['pars',
     'parameters',
     'events',
     'submodels',
     'ics',
     'initialconditions',
     'vars',
     'variables',
    ...
    ```

  


| Home | Trees | Indices | Help | PyDSTool |
| --- | --- | --- | --- | --- |

|  |  |
| --- | --- |
| Generated by Epydoc 3.0.1 on Fri May 4 15:24:06 2012 | http://epydoc.sourceforge.net |
